# Methods and Measures for Investigating Microscale Motility

**DOI:** 10.1093/icb/icad075

**Published:** 2023-06-19

**Authors:** Karen Grace Bondoc-Naumovitz, Hannah Laeverenz-Schlogelhofer, Rebecca N Poon, Alexander K Boggon, Samuel A Bentley, Dario Cortese, Kirsty Y Wan

**Affiliations:** Living Systems Institute, University of Exeter, Stocker Road, EX4 4QD, Exeter, UK; Living Systems Institute, University of Exeter, Stocker Road, EX4 4QD, Exeter, UK; Living Systems Institute, University of Exeter, Stocker Road, EX4 4QD, Exeter, UK; Living Systems Institute, University of Exeter, Stocker Road, EX4 4QD, Exeter, UK; Living Systems Institute, University of Exeter, Stocker Road, EX4 4QD, Exeter, UK; Living Systems Institute, University of Exeter, Stocker Road, EX4 4QD, Exeter, UK; Living Systems Institute, University of Exeter, Stocker Road, EX4 4QD, Exeter, UK

## Abstract

Motility is an essential factor for an organism’s survival and diversification. With the advent of novel single-cell technologies, analytical frameworks, and theoretical methods, we can begin to probe the complex lives of microscopic motile organisms and answer the intertwining biological and physical questions of how these diverse lifeforms navigate their surroundings. Herein, we summarize the main mechanisms of microscale motility and give an overview of different experimental, analytical, and mathematical methods used to study them across different scales encompassing the molecular-, individual-, to population-level. We identify transferable techniques, pressing challenges, and future directions in the field. This review can serve as a starting point for researchers who are interested in exploring and quantifying the movements of organisms in the microscale world.

## Introduction

Motility is crucial in many aspects of life, enabling organisms to find resources, evade predators, and locate or colonize suitable habitats. By employing diverse molecular motor systems, an individual organism can convert chemical energy into mechanical energy and thereby control its movement (Fletcher and Theriot 2004; Miyata et al. 2020). Swimming at the microscale is governed by fundamentally different fluid dynamics than swimming at the macroscopic length scale of our everyday experience (Purcell 1977). A major difference between motion at the micro- and macro-scales is due to the relative sizes of the *inertial* and *viscous* forces, where inertia describes the tendency of an object in motion to remain in motion, and viscosity is the frictional force that slows down an object moving in a fluid. The ratio between these two forces is called the Reynolds number (*Re*), where inertial or viscous effects dominate for high or low *Re*, respectively. For example, a human swimming in water has *Re* ≈ 10^6^, whereas a swimming *Escherichia coli* bacterium has *Re* ≈ 10^−6^. Therefore, we define microscale motility as active locomotion occurring at *Re* ≪ 1. Microscopic organisms have evolved sophisticated self-propulsion mechanisms for navigating their highly viscous environment and aiding them in activities such as photosynthesis, feeding, or reproduction, which can increase their fitness or chances of survival (Stocker and Seymour 2012).

Microbial communities are ubiquitous and underpin many biogeochemical cycles, meaning that the motility of microscopic organisms can influence food web dynamics and the structuring of ecosystems. The motility of photosynthetic (e.g., microalgae, diatoms, cyanobacteria), chemotrophic (e.g., archaea, bacteria), and heterotrophic (e.g., bacteria, ciliates, marine larvae) organisms can impact the flow of carbon and other nutrients in the food web and can affect small-scale spatial structuring of chemical and physical environmental factors (Fenchel 2002; Stocker and Seymour 2012; Worden et al. 2015; Weisse et al. 2016). With the advent of single-cell technologies and advancements in analytical and theoretical methods, we can begin to probe the complex lives of individual microscale organisms. However, bridging motility research across a continuum of physically and biologically relevant scales is daunting. Whereas population- and global-scale studies have shown how some organisms can shape large-scale ecosystem functioning through their influence on biogeochemical and nutrient cycling, studying the behavior of individuals can yield a more thorough understanding of their specific contributions. For example, *in situ* observation of marine bacterial foraging reveals specific preferences toward chemical stimuli, which can define the microscale partitioning of a community, as well as the remineralization rate of specific elements and nutrients in the ocean (Raina et al. 2022). Meanwhile, several ciliated organisms, whether free-swimming (e.g., *Paramecium*) or sessile (e.g., *Vorticella*), forage by creating feeding currents through ciliary beating. Their ciliary arrangement can influence different feeding modes (Fenchel 1982; Weisse et al. 2016) and predator evasion capabilities (Nielsen and Kiørboe 2021). Their dual role as predator and prey influences the flow of carbon and in turn the structuring of the trophic network in aquatic and terrestrial environments (Worden et al. 2015; Weisse et al. 2016; Nielsen and Kiørboe 2021).

Measuring motility at the organismal scale is often challenging due to various technical constraints (e.g., broad range of relevant length scales, fast dynamics, and requirement for specialized and expensive equipment). Ultimately, we need to ensure that the methods (i.e., experiments, analyses, and models) are appropriate, reproducible, practical, and that the interpretation of the results is accurate, insightful, and can be meaningfully associated with the biology of the organism (Berman 2018). Recent reviews have comprehensively described motility mechanisms grouping them taxonomically or based on their motility-enabling protein architectures (Miyata et al. 2020; Velho Rodrigues et al. 2021). In contrast, here, we focus on consolidating the different experimental, analytical, and mathematical methods used to study all microscale motility mechanisms across different scales from the molecular, to the individual and population levels. However, discussion on molecular techniques will be brief as extensive reviews already exist [see Beeby et al. (2020); Miyata et al. (2020), Klena and Pigino (2022); Wadhwa and Berg (2022)]. We identify commonalities between the various fields, which techniques could be transferable, and discuss common challenges and opportunities for future research.

## Mechanisms of microscale motility

**Fig. 1:**
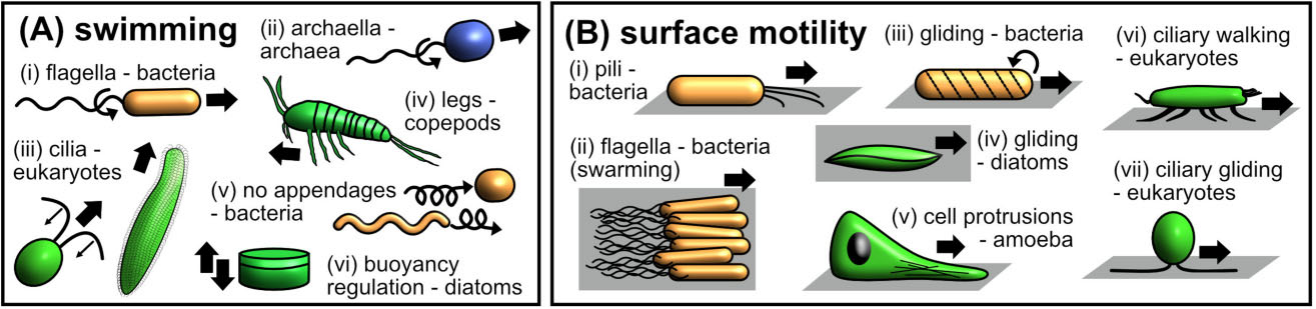
Schematic illustrations of the different microscale (**A**) swimming and (**B**) surface motility mechanisms.

In this section, we summarize the mechanisms that microscopic organisms use to propel themselves through fluids or move across surfaces (as illustrated in Fig. 1 and listed in Table 1). We note that the same style of locomotion (e.g., swimming, gliding, walking) can be achieved via different mechanisms and that the same motility apparatus can be used to achieve different types of movements. The diversity of mechanisms employed and locomotion behaviors performed by microscale organisms highlights the need to study microscale motility across different scales—from molecular mechanisms to the individual organism level and population scale.

**Table 1:** Overview of the main motility mechanisms discussed in this review

Mechanism of motility	Domain(s) of life	Example organisms
Flagella	Prokaryotes: bacteria	*E. coli, Vibrio alginolyticus*
Archaella	Prokaryotes: archaea	*Halobacterium salinarum*
Cilia	Eukaryotes: microalgae, dinoflagellates, ciliates, marine invertebrate larvae, rotifers	*Chlamydomonas reinhardtii, Dinophysis acuta, Paramecium caudatum, Platynereis dumerilii*
Swimming without appendages	Prokaryotes: bacteria	*Synechococcus* sp., *Spiroplasma citri*
Buoyancy control	Prokaryotes: bacteria; Eukaryotes: diatoms, marine larvae	*Microcystis* sp., *Anabaena* sp.; *Coscinodiscus* sp., *Acropora tenuis larvae*
Pili	Prokaryotes: bacteria, archaea	*Pseudomonas aeruginosa, Sulfolobus* sp.
Gliding	Prokaryotes: bacteria; eukaryotes: diatoms	*Flavobacterium johnsonia; Navicula* sp., *Bacillaria* sp.
Cell protrusions	Eukaryotes: amoeba	*Dictyostelium* sp., *Physarum* sp.

### Swimming

#### Life at low Reynolds number

The Reynolds number, *Re*, is a dimensionless parameter that quantifies the ratio of inertial to viscous forces acting in a moving fluid. Microscale motility occurs at “low Reynolds number”: *Re* ≪ 1, where the viscous forces experienced by such swimmers are much larger than the inertial ones. This imposes a variety of physical constraints on their motion. For example, when such a swimmer stops actively propelling itself, it will stop moving almost immediately. More subtly, such a swimmer can only propel itself by so-called “time irreversible” motions, in which a video of the swimming stroke looks different when played in reverse (Purcell 1977). The side-to-side beating of a fishtail is not time irreversible, and such a swimming stroke at low *Re* would not result in forward motion. In contrast, the “breaststroke” motion of the cilia of the low *Re* swimmer *Chlamydomonas* is time irreversible, leading to net forward motion. An effective swimming strategy under such constraints is to exploit the large difference in the viscous drag coefficient experienced by a thin rod moving parallel or perpendicular to its long axis (Becker et al. 2003). Such drag-based propulsion via the use of long slender filaments is thus common across all domains of microscopic life. Yet despite this similarity, the propulsive machinery used by archaea, bacteria, and eukaryotes (namely archaella, flagella, and cilia, respectively) are all structurally distinct, a striking example of convergent evolution (Beeby et al. 2020).

#### Flagella and archaella

The bacterial flagella and archaea’s archaella (Fig. 1A.i and ii, respectively) are both long, thin filaments (5–20 µm in length, 10–30 nm in diameter) driven by membrane-embedded rotary motors, with the former being more structurally complex than the latter. In bacteria, the rotary motor complex is powered by the ion motive force (Manson et al. 1977; Hirota et al. 1981), while in archaea, a single ATPase is responsible for torque generation (Streif et al. 2008). In both cases, the torque translates into a helical waveform and the connected passive proteinaceous filament then acts as a propeller for cell propulsion. In bacteria, this torque transduction occurs via the flagellar hook, while for archaea, the filament connects directly to the motor.

#### Cilia

**Fig. 2:**
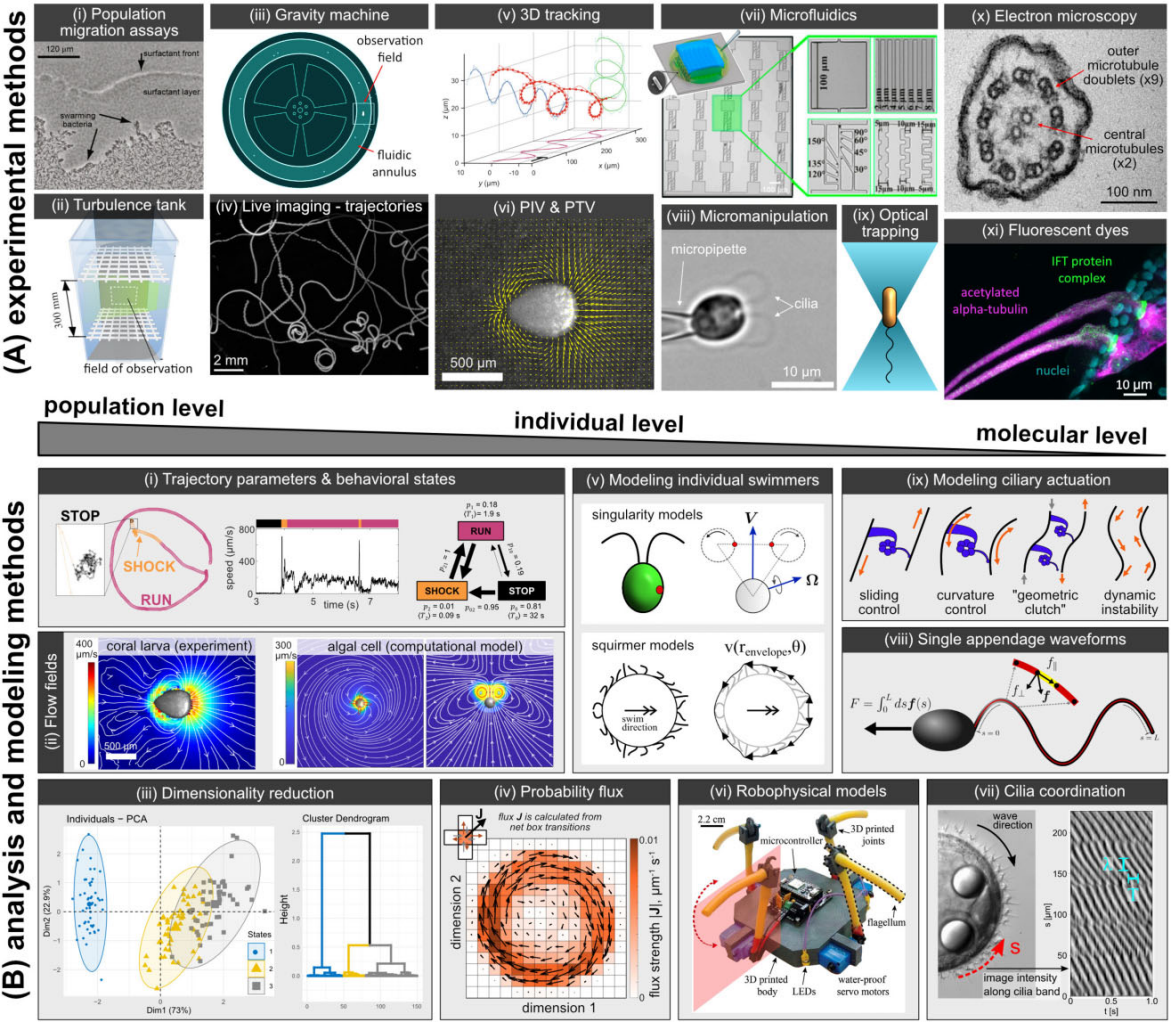
(**A**) Overview of the main experimental techniques organized by length-scale, from population- to molecular-level. (**i**) Population migration assays—the swarming behavior of a bacterial population can be observed via agar plate assays (Be'er and Ariel 2019). (**ii**) Example of a turbulence tank with two horizontal grids that oscillate to generate turbulent flow (Fagerström et al. 2022). (**iii**) Schematic of the “gravity machine” designed to track a single cell while allowing for free vertical movement (https://gravitymachine.org/). (**iv**) Live imaging, trajectories—a maximum intensity projection of jellyfish larvae trajectories recorded over a 50-s time period. (**v**) 3D tracking—a ciliate *Tetrahymena* imaged via fluorescence microscopy swims in right-handed helical tracks (Marumo et al. 2021). (**vi**) PIV & PTV—the output from running PIV on a video of a coral larva swimming through a fluid seeded with passive tracer particles. (**vii**) Microfluidics—a microfluidic chip design used to investigate bacterial swimming motility through geometries with various levels of complexity [adapted from Tokárová et al. (2021)]. (**viii**) Micromanipulation—a *Chlamydomonas* cell held by micropipette aspiration. (**ix**) Optical tweezers—a schematic of a bacterial cell held in an optical trap. (**x**) Electron microscopy—a TEM image showing the cross-section of a sperm flagellum of the hydrozoan, *Clytia hemisphaerica* (image credit: Kei Jokura). (**xi**) Fluorescent dyes—cilia of the ctenophore *Bolinopsis mikado* immunostained to show acetylated alpha-tubulin (magenta), an intraflagella transport (IFT) complex protein (green), and the nuclei (cyan) (image credit: Kei Jokura). (**B**) Diagrams illustrating the main analytical and theoretical frameworks used to study microscale motility. (**i**) Trajectory parameters and behavioral states—the locomotor behavior of the octoflagellate *Pyramimonas octopus* is classified into a trio of behavioral states based on the swimming speed. The state parameters (probabilities and expected durations) specify a unique reaction network (Bentley et al. 2022). (**ii**) Flow fields—flow fields for a coral larva, experimentally measured by PIV (Poon et al. 2022); and for an algal cell in top–down and sideways views, generated using a singularity model of the cell, and averaged over a whole beat cycle (Cortese and Wan 2022). (**iii**) Dimensionality reduction multivariate analyses can be used to reduce complex data by grouping the data in a low dimensional space through PCA and/or clustering procedures to tease apart the behavior of different organisms or assign behavioral states. (**iv**) Probability flux—the probability flux strength for trajectories of single *Chlamydomonas* cells trapped inside 40-µm diameter microfluidic droplets indicates a preferred circling direction [adapted from Bentley et al. (2022)]. (**v**) Modeling individual swimmers—using singularity methods, the cilia beating of a *Chlamydomonas* cell can be modeled by small beads constrained to rotate along circular orbits (Cortese and Wan 2021). The squirmer model approximates the hydrodynamics of a densely ciliated swimmer by specifying the fluid velocity on an envelope that covers the tips of all of the cilia. (**vi**) A robophysical model of a quadriflagellate alga (Diaz et al. 2021). (**vii**) Cilia coordination—a frame showing the metachronal wave of the ciliary band of a *P. dumerilii* larva (left-hand panel). The pixel intensity along the ciliary band gives a proxy for the cilia beat phase, and can be plotted as a 2D function of distance *s* along the band and time *t* (right-hand panel). The period of the intensity oscillations in the *s*-direction gives the wavelength *λ* and the period in the *t-*direction gives the ciliary beat period *T*. (**viii**) Single appendage waveforms—calculating the force vector per unit length on segments of a tracked cilium allows a prediction of the total force produced by the cilium. (**ix**) Modeling ciliary actuation—four primary mathematical models of ciliary actuation.

Despite appearing superficially similar, the structure of cilia (Fig. 1A.iii) is far more complex, comprising an order of magnitude more molecular components than either flagella or archaella (Beeby et al. 2020). The axoneme section of the cilium produces the characteristic bending waves used for swimming. This structure typically consists of a central microtubule pair surrounded by a ring of microtubule doublets in motile cilia (Fig. 2A.x) (Nicastro et al. 2006). Cilium bending occurs via the differential sliding of the outer doublets that is driven by dynein motors connecting neighboring microtubules (Satir 1967). These dynein motors are regularly placed along the cilium allowing force actuation along its length. The cilium can thus produce far more complicated waveforms than those achieved with flagella or archaella. Cilia are also widespread across multiple species of multicellular animals (Wan and Jékely 2020). In particular, most marine invertebrate animals have a ciliated larval stage, where the cilia contribute to swimming, sensing, and feeding (Koehl and Powell 1994; Fuchs et al. 2013; Marinković et al. 2020). Cilia in marine larvae may be localized into bands as in *P. dumerilii*, or may densely cover the entire body, as in *Nematostella* or in coral planulae larvae (Nielsen 1987; Poon et al. 2022). In contrast to unicellular organisms, these multicellular ciliated swimmers can also modify their body shapes and trajectories by muscular action. Large numbers of cilia can also bundle together to form compound cilia that can propel larger organisms at higher *Re*, for example, in ctenophores (Jokura et al. 2022).

#### Alternative swimming appendages

The above swimming mechanisms are structurally simple enough to occur in very small organisms including prokaryotes. However, many microscale animals use more complex appendages to swim (Fig. 1A.iv). Examples include the legs of multiple crustacean species such as copepods, cladocerans, and barnacles (Jiang and Kiørboe 2011; Kiørboe et al. 2014; Lenz et al. 2015; Lamont and Emlet 2018; Svetlichny et al. 2020). Being subject to similar physical constraints of low-*Re* swimming, they largely rely on the same anisotropic drag-based propulsion as cilia and flagella. However, due to their complexity, they are more structurally and anatomically diverse and so we do not discuss them further here.

#### Swimming without appendages

Swimming can also be achieved without the use of appendages, as in the bacteria *Spiroplasma* sp. and *Synechococcus* sp. (Fig. 1A.v). *Spiroplasma* sp. lacks a peptidoglycan layer on its cell wall, rendering it flexible enough to change the helicity of its body to enable swimming by kink-propagation (Shaevitz et al. 2005; Sasajima and Miyata 2021). The two ends of the cells have different handedness, and when the tapered end of the cell switches its helicity, a “kink” forms at the boundary of the axis, which then propagates to the whole cell body enabling movement (Shaevitz et al. 2005; Sasajima and Miyata 2021). Meanwhile, the swimming mechanism of *Synechococcus* sp. is less well understood. The current model suggests that it swims by forming small amplitude waves through a helical rotor powered by proton-motive forces, similarly observed in the gliding mechanism model of mollicutes (Brahamsha 1999; Ehlers and Oster 2012).

#### Buoyancy control

Some microorganisms, though nonmotile, can control their buoyancy, and thus move up and down in the water column (Fig. 1A.vi) (Boyd and Gradmann 2002). This is despite the fact that major cellular components, such as calcium carbonate or silicate shells, proteins, or carbohydrates, are inherently denser than seawater. For example, in cyanobacteria, such as *Microcystis* sp. and *Anabaena* sp., carbohydrates produced from their photosynthetic activity are used by the organism as ballast to sink to nutrient-rich deeper waters; once these reserves are expended, the cells migrate back to the surface (Kromkamp and Mur 1984). Additionally, gas vesicles, which are formed when cells are in lower, darker regions of the water column, aid in upward migration, while rising turgor pressure from the gas eventually collapses the vesicle causing the cell to sink (Pfeifer 2012; Gao et al. 2016). Similar to cyanobacteria, some planktonic diatoms (e.g., *Coscinodiscus*) actively regulate their buoyancy and can use carbohydrate ballasts (Fisher and Harrison 1996). Other means of buoyancy regulation include controlling the ionic composition of vacuoles (Boyd and Gradmann 2002) or the incorporation of silica into their cell walls (Raven and Waite 2004). Recently, it has been shown that shear stress can control cell density by eliciting an increase in cytosolic Ca^2+^ (Arrieta et al. 2020). Some ciliated marine invertebrate larvae (see section on *Cilia*) can also regulate their buoyancy using lipid reserves, which are also used as an energy source, such as in the coral *A. tenuis* (Harii et al. 2007).

### Surface motility

#### Twitching and swarming

As well as swimming in bulk fluid, many species of bacteria also move on surfaces. One form of surface motility is known as “twitching” (Fig. 1B.i). It is loosely defined as being an intermittent motion, such as that generated by the bacterial Type IV pilus, which repeatedly extends, adheres, and retracts to give a stop-and-go motion across a surface (Burrows 2012). Much of the research on twitching motility has been carried out on the pathogenic bacterial species *P. aeruginosa*, but the Type IV pilus is also found in a wide range of bacterial and archaeal species, where it may be used for functions other than twitching motility. For example, in the soil bacterium *Myxococcus xanthus*, twitching is utilized for social motility, which results in swarming, while single cells perform gliding (see section below on *Gliding*) (Mercier et al. 2020). Flagellated bacteria can exhibit a type of surface motility called swarming (Fig. 1B.ii), which is a collective motion that involves the differentiation of a vegetative (non-swarming cell) to a swarmer phenotype (Wadhwa and Berg 2022). In some bacteria, this phenotype can be hyperflagellated (e.g., *Proteus* sp.) and/or have increased cell length (e.g., *E. coli*) (Be'er and Ariel 2019).

#### Gliding

Gliding can be defined as the substrate-associated translocation of cells in the direction of their long axis without using any appendages such as cilia, flagella, or pili (Fig. 1B.iii–iv) (Henrichsen 1972). This motility mechanism has been found in distinct lineages of eubacteria (e.g., cyanobacteria, myxobacteria, bacteroidetes, mollicutes), apicomplexans, and photosynthetic unicellular eukaryotes (e.g., diatoms) highlighting the convergent evolution of unique motility machinery in different organisms (Miyata et al. 2020). We briefly summarize here the gliding mechanism of different microorganisms, more in-depth descriptions are available in the following reviews: for bacteroidetes, myxobacteria, and mollicutes (McBride 2001; Nan and Zusman 2016; Wadhwa and Berg 2022), for cyanobacteria (Wilde and Mullineaux 2015), apicomplexans (Heintzelman 2006; Frénal et al. 2017), and diatoms (Wetherbee et al. 1998; Poulsen et al. 1999). Gliding universally requires highly adhesive compounds—commonly comprised of proteins and/or polysaccharides—which are excreted onto the substrate, and are generally connected to the interior of the cell and to motor proteins that provide the motive force. The distribution of these adhesive components over the cell surface can either be in a helical pattern as in the bacteroidetes *F. johnsoniae*, the myxobacteria *M. xanthus*, and various filamentous cyanobacteria (e.g., *Oscillatoria, Phormidium uncinatum, Lyngbya* sp.); or running in parallel to the long axis of the body for mollicutes, apicomplexans, and diatoms. Interestingly, only the photosynthetic microgliders (i.e., cyanobacteria and diatoms) continuously secrete a polysaccharide-rich slime-like substance as they move. Motors for movement are also highly diverse, including modified Type IV pilus-like complexes in filamentous cyanobacteria; rotary motors powered by the proton-motive force in bacteroidetes, myxobacteria, and some mollicutes; and actin–myosin complexes in the eukaryotic microgliders (i.e., apicomplexans, diatoms).

#### Protrusion-based locomotion

Amoeboid movement (Fig. 1B.v) is perhaps one of the oldest and most well-known of all the surface motility mechanisms, with most research focusing on the social amoeba and cellular slime mold *Dictyostelium* sp. and the acellular and “many-headed” slime mold *Physarum* sp., as well as leukocytes. Organisms travel by changing their shape through protrusion and retraction of plasma membrane extensions (e.g., pseudopodia, blebs) and reversibly adhering to the surface (Lämmermann and Sixt 2009; Petrie and Yamada 2016). Forces for locomotion can be generated by actin-polymerization or hydrostatic pressure. In the former, polymerizing actin filaments can generate sufficient force to drive out membrane projections in the form of lamellipodia (flat, sheet-like branched actin filaments) or filopodia (long, thin needle-like actin projections). Meanwhile, hydrostatic pressure is formed due to actomyosin contractility. Myosin II activity triggers the formation of “blebs”: localized protrusions formed by the flow of cytosol along a pressure gradient. Bleb retraction is regulated by F-actin and actin-binding proteins, together with myosin. In *Dictyostelium* sp., both these mechanisms are observed but the preferred mode is dependent on the prevailing level of myosin II activity, with higher activity correlating with bleb formation (Lämmermann and Sixt 2009; Petrie and Yamada 2016; Raz and Schick 2022). In the case of *Physarum* sp., fan-like sheet protrusions (i.e., veins) are formed via cytoplasmic streaming governed by an actomyosin system. Fluid cytoplasm travels through the veins via propagating waves and is then converted to a more rigid version. This forms thin branch-like protrusions that can be used by the organism to explore its surroundings (Oettmeier et al. 2017; Awad et al. 2022). Protrusions can also be used for moving in a 3D environment where adhesion to surfaces is not mandatory. When cells are confined in a 3D scaffold, the retrograde flow of actomyosin is sufficient to produce friction on the walls to propel movement (Petrie and Yamada 2016). Meanwhile, in a fluid environment, cells can form “side-bumps” or sideways protrusions in the rear of the cell, which act like a paddle for swimming in the water, such as in *Dictyostelium* sp. (Van Haastert 2011).

#### Cilia-based surface motility

Cilia are not only used for swimming. Some organisms, like *Trichoplax adharens* (Smith et al. 2015; Bull et al. 2021), can use cilia to walk or crawl along surfaces. During walking, the cilia undergo a periodic stepping action, with a locomotor force generated while the cilium is in contact with the substrate. Walking motility is also observed in ciliates of the subclass *hypotrichs* (Fig. 1B.vi), which possess compound cilia called cirri on the lower surface of the cell, for example, *Euplotes* (Lueken et al. 1996; Larson et al. 2022) and *Stylonychia* (Krause et al. 2010). Each cirrus is comprised of bundles of cilia that act together as a single leg-like appendage. Another form of ciliary-driven locomotion is a type of surface gliding, most extensively studied in the microalgal species *Chlamydomonas* (Fig. 1B.vii) (Bloodgood 1988; Collingridge et al. 2013; Shih et al. 2013). Unlike other cilia-based motility mechanisms, gliding does not rely on cilia-bending movements, instead, it is powered by the intraflagellar transport mechanism, which results in longitudinal sliding movements of the ciliary membrane glycoproteins that enable the organism to move across solid surfaces (Shih et al. 2013). Several species of ciliated marine larvae also exhibit various surface motility behaviors controlled by ciliary and/or muscular action (Martin 1978; Santagata 2008).

## Current techniques for studying microscale motility

In this section, we outline the main techniques available to explore the diversity of motility mechanisms at the microscale. Due to advances in high-resolution microscopy, high-speed imaging, micromanipulation, image segmentation and tracking, machine learning, and modeling low Reynolds number fluid mechanics, the techniques available to study microscale motility are expanding, and the possibilities that come with combining the cross-disciplinary approaches promise to broaden our understanding of microscopic life. Here, our focus is on the organismal scale, that is, the experimental, analytical, and mathematical modeling approaches used to study the locomotion of individuals, but many of the techniques can be applied more broadly, for example, to study population-level dynamics. Figure 2 provides an overview of the experimental, analytical, and modeling approaches discussed below.

### Experimental methods

#### Live imaging across scales

Live imaging is the most direct approach for the experimental investigation of motility (Fig. 2A.i–ix). Whether it is imaging the waveforms of cilia, obtaining trajectories of individuals or populations, or using particle image velocimetry (PIV) to reveal the fluid flows produced by a microswimmer, capturing videos of the dynamic behavior of motile organisms is the basis for building an understanding of the mechanisms of movement, their behavioral signatures, and their response to stimuli. Most live imaging is limited in that it reduces 3D shapes and trajectories to 2D (Fig. 2A.iv). Techniques such as 3D tracking, micro-manipulation, and microfluidics enhance our ability to perform live imaging across scales. They are particularly relevant at the organismal scale and are discussed in more detail below.

In larger-scale studies, measuring behavior in populations, especially *in situ*, can be challenging due to environmental factors that cannot be controlled as carefully as in the lab (e.g., light, temperature, nutrients) and the need for specialized equipment. However, most live imaging techniques used on the organismal scale can be easily transferable to population-scale experiments in a lab setting. The most classic experimental setups are capillary assays (Adler 1966) or agar plates and other porous media (Nossal 1972; Be'er and Ariel 2019) coupled with light microscopy to track dynamic cell behaviors (Berg and Brown 1972; Taute et al. 2015; Bhattacharjee and Datta 2019) (Fig. 2A.i). Meanwhile, Couette cylinders, turbulence tanks, or the newly developed gravity machine (Fig. 2A.ii and iii) can be coupled with PIV and microscopy to study the effect of laminar shear and turbulence on swimming organisms or sinking particles (Durham et al. 2013; Krishnamurthy et al. 2020; Arnott et al. 2021). Microfluidic devices can also be used to observe the dynamic behavior of cell populations (see section below on *Microfluidics*).

#### 3D imaging

Conventional 2D imaging techniques (Fig. 2A.iv) are limited in their ability to fully resolve an organism’s movement patterns, due to the fact that organisms can change their in-focus distance (i.e., z-position) while they swim. Therefore, tracking in 3D provides unprecedented information on the motile behavior of microorganisms. A benchmark study by Berg and Brown used a tracking microscope where the sample stage moves to maintain focus on a single *E. coli* cell to determine motility changes in response to various stimuli (Berg 1971; Berg and Brown 1972). In recent years, various imaging methods (i.e., dual camera set-ups, fluorescence-based, defocused phase-contrast, and digital holographic microscopy) have been developed and improved to simultaneously track multiple cells in a 3D observation field (Wu et al. 2006; Taute et al. 2015; Bhattacharjee and Datta 2019; Marumo et al. 2021).

By using two cameras to image swimming trajectories from different orientations, the two 2D images obtained can be combined to give 3D tracks of the organism. This approach was used to study the phototactic response of the microalgae *Chlamydomonas* and *Volvox* (Drescher et al. 2009).

Fluorescence imaging relies on cells carrying fluorescent signals either by molecular labeling of cells, ingestion of fluorescent particles, or autofluorescence. Fluorescence imaging can be used to target specific features and at improved signal-to-noise ratio, and therefore, offers a range of opportunities for 3D tracking. Fluorescently labeled cells can be tracked in 3D using confocal microscopy (Bhattacharjee and Datta 2019), or a tracking microscope to keep the individual in focus (Figueroa-Morales et al. 2020). By introducing additional optical components into a conventional epifluorescence microscope, and taking advantage of the pointlike nature of fluorescent particles, Marumo et al. (2021) resolved the helical swimming of the ciliate *Tetrahymena* in 3D (Fig. 2A.v), by splitting the standard 2D image into two images such that the z-displacements of an object are transformed into relative x-displacements of the split images. However, as fluorescence relies on signal intensity, this process is limited in its spatiotemporal resolution.

Meanwhile, phase-contrast microscopy is especially useful for tracking transparent or colorless cells. When light waves pass through a cell, small changes in the phase of the light occur depending on the properties of the medium it passes through. These phase shifts are then translated into amplitude, which appears as the brightness and contrast in the output image. Defocused phase-contrast imaging is a variant of this technique, where the z-position is inferred from the out-of-focus diffraction pattern, enabling 3D tracking using a conventional phase-contrast microscope (Wu et al. 2006; Taute et al. 2015).

In recent years, the use of digital holographic microscopy (DHM) for tracking cells has also been gaining traction. As the name implies, a hologram is constructed from the interference pattern between a light beam collected from the sample and a reference beam, both of which are split from a single laser beam. The resulting image contains the sample’s phase and amplitude information, allowing a detailed reconstruction of the 3D image. DHM comes in different set-up configurations but always consists of a light source, an interferometer, a camera [normally a charged coupled device (CCD)], and a computer. Applications of DHM range from tracking particles or free-swimming cells to flow fields, and from the lab to *in situ* environments [for reviews, see Garcia-Sucerquia et al. (2006); Yu et al. (2014); Memmolo et al. (2015)].

#### PIV and PTV

Particle image velocimetry (PIV) and particle tracking velocimetry (PTV) are experimental methods for measuring the velocity field of a fluid (Fig. 2A.vi). In both methods, the fluid is seeded with passive tracer particles, and the flows are imaged at a high frame rate and resolution. Particle velocities are estimated from successive frames and used to infer the velocity field of the fluid (Adrian 1991). These methods allow quantification of the flows induced by a microswimmer in a fluid (Fig. 2B.ii), which has various applications. A direct measurement of the flow field around a microswimmer can be used to compare the measured swimming behavior with simple physical models (see section below on *Modeling individual microswimmers*). Such measurements of the flow fields around swimming algae such as *Volvox* and *Chlamydomonas* have been used to construct appropriate flow-singularity models for those organisms (Drescher et al. 2010). In other species, it can also be used to quantify feeding flows and clearance rates (Nielsen and Kiørboe 2015).

Basic implementations of PIV measure the flow field in a 2D slice. Traditionally, imaging is limited to a single plane by illuminating the fluid with a laser light-sheet so that only the in-plane particles are visible. However, it is difficult to produce a sufficiently thin light-sheet to use this method at the high magnifications necessary to image objects such as microswimmers, leading to the recent development of “microPIV” (Lee and Kim 2009). These methods exploit the finite focal depth of a high-magnification objective, which naturally restricts the imaging plane. Micro-PIV can then be readily performed to measure flows produced by motile organisms if the microscope is already equipped with a high-speed camera. The only additional component required is a suitable choice of tracer particles, seeded into the flow at an appropriate density. The optimal diameter of the particles depends on the size of the organism in question and the magnification being used, and in practice is normally between 0.3 and 5 µm (Drescher et al. 2010; Gemmell et al. 2014; Nielsen and Kiørboe 2021). Polystyrene microspheres are commonly used. However, the presence of such “artificial” materials can affect the natural behavior of microswimmers, so biological particles such as yeast, microscopic droplets in milk, or non-motile or slow-swimming microalgae can also be used, thus limiting the effect on the behavior (Kowalczyk et al. 2007; Gemmell et al. 2014; Wandel and Holzman 2022). The seeding density depends on the magnification and the average flow speed, and whether the data will be analysed by image correlations (PIV) or by particle tracking (PTV). “Rules of thumb” for choosing appropriate parameters can be found in works by Keane and Adrian (1990), Melling (1997), and Scharnowski and Kähler (2020). There are several toolboxes available for performing the analysis, such as the MATLAB PIVlab toolbox (Thielicke and Stamhuis 2014), or the Python openPIV package (Liberzon et al. 2021).

PIV and PTV use similar experimental setups but different analysis methods to calculate the flow field. In PIV, each frame is divided into small “windows” and the image correlation between successive frames is computed for each window, giving a velocity field that is evenly sampled in time and space. It performs best when the particles can be homogeneously seeded throughout the flow, at a sufficiently high density. In PTV, individual particles are tracked as they move through the fluid. PTV can give much higher spatial resolution than PIV, which is limited by the size of the interrogation windows. However, it also poses the challenge of accurate individual-particle detection (Ohmi and Li 2000).

In the basic experimental setup for both methods, only the in-plane velocity can be measured, but setups such as scanning light-sheet microscopy (Brücker 1995), holography (Pu and Meng 2000), 3D PTV (Virant and Dracos 1997), and tomographic PIV (Elsinga et al. 2006) can measure 3D flow velocities. For more detail and background, we refer the reader to a comprehensive reference book by Raffel et al. (2018) and a review of the development of these methods over the past decades by Adrian (2005).

#### Microfluidics

Microfluidics involves the manipulation of fluids at volumes of micro-liters and smaller, using micron-sized channels (Fig. 2A.vii). It has grown rapidly in recent decades due to its potent biochemical and medical applications, such as conducting immunoassays (Weibel et al. 2005) or performing single-cell DNA barcoding on a large scale (Zillionis et al. 2016). It is also a flexible and powerful technique for studying motility at the microscale (Son et al. 2015).

Microfluidic chips can be designed to perform other functions such as mixing fluids (Lee et al. 2011), applying chemical gradients using permeable membranes (de Jong et al. 2006), or altering surface characteristics (e.g., hydrophobicity) via fabrication with particular chemical coatings (Raj M and Chakraborty 2020). In addition, droplet microfluidics can be used to confine cells further by trapping them in water-in-oil emulsions (Bentley et al. 2022). The ease of manufacture of chips allows for successive improvement of designs for rapid prototyping (Zheng et al. 2012, 2013, 2014) or small design modifications to compare slight variations in environments (Ostapenko et al. 2018).

Microfluidic techniques have allowed the study of motility in such diverse microswimmers as bacteria (Kalinin et al. 2009, 2010), unicellular algae (Ostapenko et al. 2018), and mammalian sperm cells (Samuel et al. 2018). The natural local environments of microswimmers are heterogeneous; they can be open or highly confined (Tokárová et al. 2021) and display complex boundaries and solid-fluid interfaces (Théry et al. 2021), such as the porous soil in which the motile microalga *C. reinhardtii* lives (Kreis et al. 2018) or the mammalian fallopian tube that sperm cells swim through (Nosrati et al. 2017). Microfluidics is thus ideal for creating experimental environments that resemble the natural environments motile microorganisms must navigate. Both individual cells and large populations can be easily observed when placed within such a device, and in combination with microscopy and cell tracking, behavior can then be measured and analyzed using trajectory data for either individual cells (Ostapenko et al. 2018; Bentley et al. 2022) or larger populations (Kalinin et al. 2010; Rusconi et al. 2014). This allows the observation of motility across spatial scales, which can give insights into the heterogeneity of behavior across the population and how individual organisms interact with their conspecifics.

One characteristic of microfluidic devices, which is key to their usefulness, is that at micron scales *Re* is low and so fluid flow is laminar, and thus (to some extent) predictable and easier to analyze (Schuster et al. 2003; Samuel et al. 2018). However, a drawback is that they are most useful for studying swimming organisms; studying other forms of motility such as surface-bound gliding motility requires careful consideration of the chemical and physical properties of the different surfaces (e.g., glass, PDMS) involved (Ducret et al. 2013).

Unicellular swimming algae, primarily the model species *C. reinhardtii*, are commonly used in microswimmer research due to the structural and functional similarity of their cilia to those present in mammals such as humans. Microfluidic devices have been used to study the interaction of swimming *C. reinhardtii* and its cilia with surfaces (Kantsler et al. 2013; Contino et al. 2015), the effect of boundary curvature on cell location and concentration (Ostapenko et al. 2018), and the cell's response to light stimuli (Bentley et al. 2022).

Microfluidics has also been used in a range of experiments studying bacterial motility. It is a powerful tool for studying bacterial chemotaxis since stable and reliable chemical gradients can be formed by fluid flow. For example, agarose gel can be used within microfluidic devices to produce a barrier to the fluid that allows the diffusion of small molecules across it, generating a stable chemical gradient in an environment. These gradated environments have proven very useful for probing behavior and understanding the chemical pathways of tactic behavior, especially for *E. coli* (Ahmed et al. 2010; Colin and Sourjik 2017).

Due to its ability to create highly controlled environments, microfluidics has been used in experiments to test predictions made by multi-scale theoretical models (Kalinin et al. 2009; Cammann et al. 2021; Tokárová et al. 2021). For example, the work of Kalinin et al. (2009) confirmed that *E. coli* has high sensitivity toward gradients of the chemoattractant amino acids, *α*-methyl-dl-aspartate and L-Serine. Follow-up work demonstrated how *E. coli* responds to multiple chemical gradients, which are common in natural environments but difficult to produce consistently *in vitro* (Kalinin et al. 2010).

The mechanics of bacterial navigation and motility can also be readily studied in a microfluidic device. For example, the work of Tokárová et al. (2021) focused on the effect of high levels of confinement and boundary encounters in five distinct bacterial species. The authors then compared the experimental cell trajectories with theoretical models of how bacterial wall interactions vary with cell size and flagellar arrangement. This work highlighted the remarkable potential of microfluidics to reveal novel behaviors in microswimmers, such as helical motion in highly confined channels. The work of Binz et al. (2010) had a narrower focus but, in addition to observing higher cell velocities in *Serratia marcescens* under confinement than in open field experiments, also demonstrated a similar zig-zagging (or perhaps helical) behavior while in highly confined channels.

#### Micromanipulation

Free-swimming individuals are often challenging to image at high magnification over long time periods. To observe the detailed waveforms of motile appendages and study long-term behavioral characteristics, the organism’s body can be held fixed by micropipette aspiration (Fig. 2A.viii) [e.g., see Rüffer and Nultsch (1990); Brumley et al. (2014); Wan et al. (2014)]. Micropipettes are typically fabricated from glass capillaries using a micropipette puller. The inner and outer diameters of the micropipette must be carefully chosen such that it creates the necessary suction force while not sucking the individual too far into the pipette. Fire polishing the tip helps to create rounded edges to minimize the risk of damaging the organism (Oesterle and Instruments 2018).

Micromanipulation tools can also be used to study how microscale organisms respond to stimuli. For example, a small glass stylus or microneedle can be used to apply a mechanical stimulus at a precise location (Ogura and Machemer 1980; Krause et al. 2010), micropipettes can introduce a localized flow (Wan and Goldstein 2014), and cells held on a micropipette can be exposed to different controlled flow environments by holding them inside a microfluidic channel (Klindt et al. 2016).

When properly calibrated, micropipettes can also be used as force sensors by measuring the pipette deflections at high spatial and temporal resolution (Schulman et al. 2014; Böddeker et al. 2020). For example, the forces produced by the beating cilia of *Chlamydomonas* were measured by aspirating a cell to the end of a highly flexible double-L-shaped micropipette, which acts as a calibrated dynamic force cantilever (Böddeker et al. 2020).

Micromanipulation techniques also enable electrophysiological experiments of microswimmers, for example, to investigate the bioelectric control of the beat direction, waveform, and frequency of motile cilia (Machemer 1974; Machemer and Sugino 1989; Elices et al. 2023). This typically involves inserting a glass electrode into an individual cell and measuring its membrane potential, either to determine the organism’s inherent electrical properties and spontaneous activity or reveal how the membrane potential responds to stimuli (e.g., current injection or mechanical stimulation). Electrophysiological experiments have been most extensively applied to study the ion channel properties and bioelectric control of ciliary beating in *Paramecium* (Brette 2021), but have also been performed with other ciliate species [e.g., Lueken et al. (1996); Hennessey and Kuruvilla (2000); Krause et al. (2010); Echevarria et al. (2016)] and microalgae (Harz and Hegemann 1991). These studies demonstrate the importance of the membrane potential in controlling motility and show that ions such as Ca^2+^, K^+^, and Na^2+^ play a central role in controlling the movements of motile appendages and coordinating an organism’s response to environmental stimuli.

Bioelectric signaling can also be studied by imaging the dynamics of calcium and voltage-sensitive dyes using fluorescence microscopy (Grienberger and Konnerth 2012; Xu et al. 2017). While microelectrode recordings are typically more accurate and can achieve a higher time resolution, fluorescence imaging minimally disrupts an organism’s behavior and does not require it to be immobilized. Fluorescent indicators of bioelectric activity can be genetically encoded (Randel et al. 2014; Xu et al. 2017), or introduced into the organism by incubating it with the relevant dye (Alvarez et al. 2012), biolistic loading (Collingridge et al. 2013), or delivered via microinjection directly into the individual (Iwadate and Suzaki 2004).

Microinjection is a technique in which a sharp micropipette is loaded with a chemical of interest and inserted into the organism for the intracellular delivery of fluorescent dyes or precise chemical stimuli. It is typically combined with microscopy to image the fluorescence signal and/or motility dynamics (Tamm and Terasaki 1994; Iwadate and Suzaki 2004). It has also been successfully performed in conjunction with electrophysiology experiments (Nakaoka and Machemer 1990; Pernberg and Machemer 1995). Microinjection techniques have been used to introduce calcium indicators into the cytoplasm and into cilia to measure the calcium signaling dynamics associated with motility behaviors in, for example, the ciliates *Paramecium* and *Didinium* (Pernberg and Machemer 1995; Iwadate et al. 1997), and the ctenophore *Mnemiopsis* (Tamm and Terasaki 1994). It has also been used to control the intracellular concentrations of calcium and cyclic nucleotides to study their effect on ciliary beating in *Paramecium* (Nakaoka and Machemer 1990; Iwadate and Nakaoka 2008).

A different approach to micromanipulation is optical trapping (also known as optical tweezers), which uses highly focused laser light to generate piconewton forces able to manipulate objects that are typically nano- or micro-scale in size (Fig. 2A.ix) (Favre-Bulle et al. 2019). Particularly relevant to the study of motility, optical trapping can be used to actively position and probe biological systems (including single molecules, organelles, and cells) (Ashkin et al. 1987; Favre-Bulle et al. 2019), which enables detailed observation of motility behaviors and dynamics (Min et al. 2009). Optical tweezers have also been used to measure the swimming forces generated by, for example, sperm (Nascimento et al. 2008) and *E. coli* (Armstrong et al. 2020).

Contact-less micromanipulation can also be achieved using acoustic traps (Ozcelik et al. 2018; Meng et al. 2019) and magnetic tweezers (De Vlaminck and Dekker 2012; Kilinc and Lee 2014), which are analogous to optical tweezers but use sound waves and magnetic fields, respectively, to generate the trapping force, instead of light. Example studies include the use of acoustic traps to characterize cell motility phenotypes (Kim et al. 2019; Rode et al. 2022) and to study the behaviour of active matter under confinement (Takatori et al. 2016). Magnetic tweezers can be used to generate forces and torques to measure the mechanical properties of a biological sample (e.g., a single-molecule or cell) by attaching magnetic beads to it and then manipulating the beads using a magnetic field (Neuman and Nagy 2008). For example, magnetic tweezers were used to measure the maximum torque produced by the flagellar motor of *E. coli* (Wang et al. 2022).

#### Molecular structure

To understand the motility mechanisms available to an organism, it can be informative to study the structure of the motility apparatus on a molecular level. Here, we only briefly discuss the molecular basis of motility, for more details, see, for example, the recent reviews Beeby et al. (2020), Klena and Pigino (2022), and Wadhwa and Berg (2022). Electron microscopy (EM) and confocal imaging can resolve these structures in great detail [e.g., when applied to the model organism *Paramecium*, see Aubusson-Fleury et al. (2015)]. An organism’s motility is determined both by *which* structures the organism possesses, and *how* they are used. For example, the maximum speed of a multiciliated organism will depend both on the density of the cilia and the frequency at which the cilia beat. Due to limited image resolution and complications due to fast-beating cilia, it is often difficult to measure cilia spacing by live imaging, and specimens must be fixed and imaged, normally by EM, to obtain such structural information. It is possible, though difficult, to fix samples for EM instantaneously, giving a “snapshot” of the cilia behavior during normal swimming (Larsen and Satir 1991). Electron microscopy, particularly transmission electron microscopy (TEM) and cryo-EM, has also helped to reveal the internal molecular structures of motile appendages (Fig. 2A.x). Such studies have been instrumental in showing that the locomotor force is generated along the whole length of a cilium, whereas for flagella and archaella, the force is generated by molecular motors at the base (Beeby et al. 2020; Wadhwa and Berg 2022).

Additionally, various structures in a specimen can be stained via appropriate antibody preparations and visualized using confocal microscopy (Fig. 2A.xi). For example, immunostaining revealed the role of striated fibers in promoting basal body connections in the ciliate *Tetrahymena* (Soh et al. 2019) and cilia rootlets can be stained to show their preferred beating direction (Bengueddach et al. 2017). Recently, new sample preparation methods have led to the development of “expansion microscopy,” which enables nanoscale resolution imaging with standard fluorescence microscopy by physically expanding fluorescently labeled fixed samples (Gambarotto et al. 2019; Wassie et al. 2019). Most fluorescence imaging is limited in that it requires fixed samples; however, live imaging of the cytoskeleton can be achieved with specific fluorescent probes that stain the relevant protein filaments (e.g., tubulin or actin) (Lukinavičius et al. 2014).

### Analysis and modeling methods

We now present an overview of analysis and modeling procedures (Fig. 2B) that can be used to understand the vast experimental data sets produced by the different methods described above.

#### Trajectory analysis

Videos provide observational evidence of how microscopic organisms move and how they respond to environmental stimuli. When observing an organism’s movements, we are often faced with the questions—how can this be quantified? What are the meaningful parameters that describe its motion? What is the best representation of the organism’s behaviors? How can quantitative techniques enrich our understanding? Can they reveal hidden dynamics not immediately obvious from observation alone?

Once images are acquired, the first step toward quantifying the motility usually involves some form of image segmentation, detection, and tracking to obtain trajectories. Various algorithms have been developed for these processes and a variety of commercial and open-source image processing and tracking programs are available (e.g., Imaris, Icy, Livecyte, TrackMate, TrakEM2, CellTrack, CellMissy, etc.), commonly automated for high-throughput processing with multiple user interfaces for different use cases. For more information on algorithms and available tracking software, see Meijering et al. (2012), Chenouard et al. (2014), Ulman et al. (2017), BoquetPujadas et al. (2021), and Emami et al. (2021). Depending on the purpose of the study and the organism, tracking algorithms can follow the centroid position, the organism’s shape, or the appendage waveform. Among the available image processing platforms, the open-source program ImageJ is extensively used. In particular, its TrackMate toolkit provides effective feature extraction, segmentation, and tracking algorithms for obtaining trajectories, as well as some derived motility parameters (Tinevez et al. 2017; Ershov et al. 2022).

From trajectories, we can obtain a coarse-grained description of movement characteristics (Fig. 2B.i). The most common track parameters are speed, turning angle, angular velocity, path curvature, and location (spatial distribution). Other related characteristics such as mean square displacement, and persistence measures (i.e., linearity, confinement ratio, asphericity, displacement ratio, etc.) can also give information about the organism’s behavior. The most commonly measured motility parameters are outlined in various resources (Meijering et al. 2012; Svensson et al. 2018).

Motility parameters can be used to define the baseline behavior of organisms as well as their response to different environmental stimuli (Berg and Brown 1972; Bentley et al. 2022; Echigoya et al. 2022). Furthermore, they can also be used to designate behavioral states (see section below on *Behavioral states*), or as training data for machine learning, which can aid in high-throughput analysis for phenotyping behavior of cell populations (Choi et al. 2021). Various frameworks have been developed in this regard, which use several multivariate analyses or regression procedures to simplify the motility space prior to clustering or classification (see section below on *Dimensionality reduction and clustering techniques*).

#### Differential dynamic microscopy

Differential dynamic microscopy (DDM) is a high-throughput analysis framework that allows the identification of population-averaged motility parameters in 3D without individually resolving the objects (e.g., cells, cilia) in question. It relies on the decorrelation of images in time to describe the dynamics of the system (Cerbino and Trappe 2008). The principal output of DDM is the differential intensity correlation function (DICF), which measures the average correlation between any two images separated by a given time interval. Assuming the objects have isotropic motion and the intensity fluctuations in the images are proportional to fluctuations in the number density of objects, the DICF can be related to the intermediate scattering function (ISF) (Cerbino and Trappe 2008). The ISF can be fitted to the data to extract the system dynamics. In some simple cases, the ISF has an analytical form, alternatively, ISF models can be constructed (Croze et al. 2019). DDM complements tracking approaches as the ISF models are built from a knowledge of the individual dynamics typically obtained from tracking studies. For example, DDM has been used to obtain the diffusion coefficient of Brownian spheres (Cerbino and Trappe 2008) and the swimming dynamics of *E. coli* and *C. reinhardtii* cells (Martinez et al. 2012). Extensions to this classical formulation of DDM include multiscale DDM used to extract the wavelength and direction of metachronal waves as well as the cilia beat frequency in ciliated tissues (Feriani et al. 2017).

#### Behavioral states

From microscopy observations and trajectories, we often find that an organism’s movements can be categorized into a small set of behavioral states, with each state associated with a characteristic or stereotyped mode of locomotion, analogous to the walk, trot, and gallop gaits of a horse. Each gait typically involves a different mode of actuation in the motility apparatus. This type of description has been famously applied to the movements of *E. coli*, which can be categorized into two states—periods of straight swimming when the flagella are bundled together are called “runs,” interspersed with active reorientations called “tumbles” that occur when flagella unbundle (Berg and Brown 1972; Wadhwa and Berg 2022). Other motility strategies described using the behavioral states approach include “run-reverse-flick” in the bacterium *V. alginolyticus* (Son et al. 2013; Wadhwa and Berg 2022), a eukaryotic version of “run-and-tumble” in *C. reinhardtii* (Polin et al. 2009; Bentley et al. 2022), “run-stop-shock” in the microalga *P. octopus* (Wan and Goldstein 2018; Bentley et al. 2022), “helical-spinning-polygonal” swimming in *Euglena gracilis* (Tsang et al. 2018), “roaming-and-dwelling” in the ciliate *Tetrahymena* (Jordan et al. 2013), and the “droplet-cone-trumpet” states in the ciliate *Stentor coeruleus* (Echigoya et al. 2022).

Behavioral states are typically classified using trajectory parameters such as speed, acceleration, track curvature, or organism shape. This generally requires the researcher to first asses the movement characteristics of the particular species and identify a subset of characteristic gaits. Setting thresholds for relevant parameters is often a suitable baseline approach to classifying states. Alternatively, clustering and other dimensionality reduction techniques (see section below on *Dimensionality reduction and clustering techniques*) have also been used to identify behavioral states (Echigoya et al. 2022; Larson et al. 2022), which minimizes any potential researcher bias. With advances in machine learning, it may soon be possible to find more unsupervised methods for identifying a discrete number of states from trajectories of any given organism, without the need to create custom algorithms (Choi et al. 2021).

By analyzing motility through the lens of behavioral states, we obtain a low-dimensional description that can be useful in comparing the different strategies microscopic organisms employ to effectively navigate their surroundings. Once a trajectory is decomposed into a series of states, as well as characterizing the properties of the different states, we can use the discrete time series to specify a network, analogous to a chemical reaction network, with state probabilities, expected state durations, and transition rates between the different states (Fig. 2B.i). This discrete state representation allows us to quantify how sub-cellular dynamics change over time or in response to environmental cues (Wan and Goldstein 2018; Bentley et al. 2022; Echigoya et al. 2022).

#### Dimensionality reduction and clustering techniques

The high-speed and long-term imaging required to capture dynamic motility behaviors often produces complex high-dimensional data sets, whereas locomotor strategies are often highly stereotyped and low-dimensional. This is a recognized challenge in neuroethological studies of animal behavior, and recent advances in quantitative analysis frameworks and machine learning enable low-dimensional descriptions of organism behavior to be achieved (Berman 2018; Datta et al. 2019). Approaches used in animal behavior research can be usefully applied to study motility in microscopic organisms, since in both cases, the raw data often consists of movement trajectories or videos of the individual’s body postures.

Standard multivariate analyses can be powerful tools for understanding and visualizing the multi-dimensionality of large datasets produced by track analysis. Mapping the data in a lower-dimensional space through principal component analysis (PCA), t-SNE (t-distributed stochastic neighbor embedding), or UMAP (Uniform Manifold Approximation and Projection) reduces the complexity of datasets and removes the noise while preserving important characteristics of the original data. Clustering techniques such as hierarchical clustering on principal components (HCPC) and *k*-means clustering assess the robustness of the grouping, the results of which are commonly depicted as a dendrogram (Fig. 2B.iii). Despite the power of dimensionality reduction techniques, applications in microscale motility studies are less common. Examples include identifying the basic waveforms and fluid interactions that drive propulsion in sperm cells (Ma et al. 2014; Werner et al. 2014; Ishimoto et al. 2017), assessing the possible number of states/gaits of a moving organism (Werner et al. 2014; Kimmel et al. 2018), and phenotyping motility of a population (Kimmel et al. 2018; Heryanto et al. 2021; Echigoya et al. 2022; Xin et al. 2022). These studies highlight the potential of this technique in understanding the complex landscape of motility both at individual- and population-level, which is akin to its use in animal behavioral studies (Berman 2018; Datta et al. 2019).

#### Probability flux

To further investigate how motility mechanisms and stochastic behaviors are associated with low-dimensional characteristics within high-dimensional parameter spaces, the concept of probability flux from statistical physics provides a useful measure to account for the arrow of time, characterize non-equilibrium dynamics, and reveal hidden patterns in motility behaviors. Once a parameter space of interest has been identified, the probability flux is a vector field within this parameter space. The probability flux has a heading and a strength at each position, and reveals whether there are any preferred pathways through the chosen parameter space. Probability flux analysis was first introduced by Battle et al. (2016) to study the period beating dynamics of an isolated beating cilium of *C. reinhardtii* in a phase space representing the cilium shapes. The approach has since been applied to analyze the long-time trajectories of individual microswimmers in confined physical geometries, revealing the emergence of self-organized flux loops (Fig. 2B.iv) (Cammann et al. 2021; Bentley et al. 2022).

#### Modeling individual microswimmers

Various mathematical descriptions have been investigated for a wide range of microswimmers. One main benefit of using a simple mathematical model to describe a swimmer is that it can be used to test the behavior and response of a swimmer to environments and conditions that may be difficult or impossible to create experimentally. Additionally, models give insight into the most important features of the organism’s swimming—if certain aspects of the real swimmer are irrelevant to the model, then that implies they are irrelevant to the swimming mechanism. Finally, models allow comparisons between the swimming of multiple organisms that can be described by the same model. Here, we consider two main classes of model: singularity methods, and squirmer models (Fig. 2B.v). Computational fluid dynamics can be used to implement more detailed and realistic swimmer models (Scherr et al. 2015), but this is beyond the scope of this review.

##### Singularity methods

In the *Re* ≪ 1 regime typical of microswimming, the Navier–Stokes equations governing the velocity of an incompressible Newtonian fluid reduce to the so-called Stokes equations (Lauga and Powers 2009). Singularity models are approximate solutions of the Stokes equations in the presence of a source of disturbance, such as a microswimmer in a given geometrical configuration or underlying flow.

Singularity models are a form of multipole expansion, analogous to those used in electromagnetism and gravitational physics to express the force fields at points distant from their sources. In the case of microswimmers, the source of the fluid velocity field is the swimmer, whose long-range effect on the fluid can be approximated by the superposition of terms that correspond to different configurations of point sources. Examples include the field generated by a single point-force or monopole called a stokeslet, that generated by a force dipole, which can be axisymmetric or composed of a symmetric (stresslet) and antisymmetric (rotlet) part, or a quadrupole, and so on. These solutions are called singularities because the velocity field tends to infinity at the exact location of the source. Whether a term in this series is present in a specific model depends on the symmetries of the microswimmer and the obstacles (e.g., walls, other swimmers) surrounding it (Blake and Chwang 1974). Although the simplest singularity solution to the Stokes equations is the stokeslet, most microswimmers are best modeled by solutions with zero net force, such as dipoles (Pedley and Kessler 1990). This is because the swimming mechanism is typically due to internal forces, rather than external ones. Most swimming microorganisms are also not subject to external torques, which limits the mathematical expression of the dipole term to its symmetric part—the stresslet. The flows induced by a swimming bacterium such as *E.coli* is indeed well described by a singularity model including only a stresslet term (Drescher et al. 2011).

The two point-forces composing a stresslet can either point toward the interior of the swimmer or toward the surrounding fluid. In the first case, the microswimmer is called a “puller” and swims by using its appendages or shape to pull the fluid in front of itself toward its own body and redirect it sideways. In contrast, a “pusher” swimmer pushes the surrounding fluid away from itself at the back, thus swimming body-first. Typical pullers include *Chlamydomonas* and many flagellated algae, while typical pushers are bacteria such as *E. coli*. Singularity models have been very successful at modeling the flows induced by cells swimming in a boundless fluid or near obstacles (Berke et al. 2008; Drescher et al. 2010), and also for understanding more complex three-dimensional behaviors such as super-helical navigation and phototaxis (Fig. 2B.ii) (Cortese and Wan 2021).

##### Squirmer model

The squirmer model of Lighthill and Blake (Lighthill 1952; Blake 1971) is used to model multiciliated swimmers, which have cilia densely covering a large proportion of their bodies. It would be very computationally expensive to simulate or solve a model that includes such a large number of individual cilia. As such, the squirmer model approximates the individual cilia by a continuous, approximately spherical, “envelope” that covers the tips of all of the cilia. It is then more straightforward to solve the fluid equations to find the flow resulting from the movement of this envelope. However, it is important, and sometimes not trivial, to choose an appropriate shape and speed for the cilia envelope. This is informed by the length and beat pattern of the individual cilia, and also any features of their global coordination (see section below on *Cilia coordination and metachronal wave analysis*). The spherical colonial alga *Volvox* has been extensively modeled as a spherical squirmer (Pedley 2016). The squirmer model has also been used to study the behavior of multilicated swimmers near boundaries (Ishimoto and Gaffney 2013), to compare the efficiencies of different forms of metachronal coordination (Blake 1971), and has been extended to non-spherical body shapes (Theers et al. 2016; Zantop and Stark 2020).

#### Physical modeling

Microswimmers can also be modeled using macroscale physical models. The physics of the low *Re* regime can be recovered at this larger scale by choosing a fluid of suitable density and viscosity to give an *Re* comparable to that of a microswimmer in water. In a similar way to computational models, physical models use a “bottom-up” approach to study the system, by implementing the minimal number of components necessary to reproduce the basic swimming behavior of the organism. For example, a minimal robophysical model of a quadriflagellate swimmer (Fig. 2B.vi) can successfully reproduce the relationship between gait and swimming performance observed in live microalgae (Diaz et al. 2021). Artificial ciliary arrays can also be programed to perform metachronal waves and used to investigate the effect of different wave parameters on various properties of the fluid flow (Dong et al. 2020).

#### Cilia coordination/metachronal wave analysis

Multicilated organisms overwhelmingly display some degree of coordination in their ciliary beating. To quantify and analyze the coordination dynamics, it is necessary to extract the phase of the cilia from the video data. When the cilia are widely spaced, the individual cilia can be tracked from video data. Such analysis of *Chlamydomonas* has shown that the dynamics of its two cilia are more complex than simple synchronous beating (Wan et al. 2014). Many organisms, from unicellular ciliates such as *Paramecium*, the colonial alga *Volvox*, through to larvae of marine invertebrates such as *Platynereis*, have large numbers of cilia, distributed all over their body, or localized into ciliary bands (Párducz 1967; Brumley et al. 2015; Marinković et al. 2020). Such arrays of multiple cilia usually coordinate into metachronal waves, where cilia organize into synchronously beating rows, with a constant offset in the beat phase between neighboring rows. In such multiciliated systems, the beat phase must be inferred from image intensity fluctuations within carefully chosen windows, due to the high cilia density. For example, in a video of a beating ciliary array, periodic oscillations of the intensity over the array give a proxy for the beat phase at that point (Fig. 2B.vii) (Wan et al. 2019). Where the imaging resolution is insufficient to resolve the cilia themselves, local variations in the fluid flow velocity can be used instead as a proxy for the beat phase (Brumley et al. 2015; Poon et al. 2022). Video data can thus be analyzed to find parameters such as wavelength, frequency, direction, and coordination length- and time-scales (Ringers et al. 2023). Measuring such parameters in experimental systems allows comparison with computational metachronal wave models such as those of Meng et al. (2021) and Solovev and Friedrich (2022), and can inform simulations of the cilia-driven swimming of organisms (Blake 1971; Ito et al. 2019). Finally, metachronal coordination is not limited to ciliated swimmers and is observed in a broad range of organisms, for example, in the ctenes of ctenophores and the pleopods (“swimming legs”) of shrimp (Byron et al. 2021).

#### Cilia tracking, waveform analysis, and modeling

Full appendage tracking provides data for comparison with theoretical models, having been used to study the regulation of dynein motor actuation in cilia (Sartori et al. 2016). The extracted waveforms can also be used directly to predict the swimming behavior of a simulated microorganism (Gallagher and Smith 2018).

Beyond characterizing the dynamics of appendages, waveform tracking can be used to elucidate the force generated by these appendages (Fig. 2B.viii). This is relevant for simple models of how locomotion is achieved in single-cell organisms and provides an approximation for the forces expected from bottom-up models of ciliary or flagellar molecular propulsion (Johnson and Brokaw 1979).

The simplest theoretical framework with which to determine the force produced by an actuated filament at a low *Re* number is that of local drag theory or resistive force theory (RFT) (Gray and Hancock 1955). In this approach, the filament can be modeled as a series of straight rods that experience a uniform force per unit length when driven by some external force, which in the case of microswimmers will be the propulsive machinery of the cilia, flagella, or archaella. Based on this approximation, the fluid flow due to a deforming filament is replaced by that of a line of stokeslets (the fluid flow due to a point force) of appropriate strengths. Using this approach, we can obtain an analytical form for the force produced by a moving filament in terms of the motion of the individual rods into which the filament is separated. This formulation is then ideally suited to analyze a tracked appendage that is already separated into discrete elements by the tracking. As such, RFT is regularly used as a method by which to evaluate the propulsive forces generated by flagellated and ciliated microorganisms (Gray and Hancock 1955; Friedrich et al. 2010; Velho Rodrigues et al. 2021).

RFT does, however, have several critical limitations. The theory does not account for long-range hydrodynamic interactions, end effects at the filament tip, or the interaction between the cell body and the filament when used to predict the swimming dynamics of microorganisms. To account for these, an alternative theory was developed called slender body theory (Hancock 1953).

Slender body theory (SBT) differs from RFT by taking into account the (decaying) effect on the flow at a given point along the filament from points at increasing distances along the filament. SBT has received multiple rigorous mathematical treatments [see references in Lauga and Powers (2009)] but a more physically intuitive description was given by Lighthill (1976). His description of SBT involves modeling the flow at a point, *s*_0_, on the filament as a superposition of the flow from the “inner” and “outer” problems. In the inner problem, the filament in the region near *s*_0_ is modeled as a combination of stokeslets and source dipoles. In the outer problem, the filament further from *s*_0_ is treated again as a line of stokeslets, because the dipole flow field decays spatially much faster than that of a stokeslet. This model captures the essence of SBT and makes it clear that it incorporates more fully the impact of interactions between different parts of a filament.

It is worth noting that the improved accuracy of SBT does come with a computational cost. As such, it is necessary to identify whether RFT remains an appropriate modeling choice for the problem under consideration, see Johnson and Brokaw (1979) and Walker et al. (2019).

#### Modeling ciliary actuation

Delving further into the swimming mechanisms of individual cells, considerable attention has been given to understanding the actuation of cilia. The propulsive machines underlying cilium movement are the hundreds of dynein motors working in concert to bend the axoneme structure (Satir 1967). How dynein activity is regulated to set up regular beating patterns has been the subject of several modeling approaches (Fig. 2B.ix) with no single model gaining a consensus in the field.

Three main models focusing on individual dynein activity regulation have been proposed. In each model, the activity of the dyneins on one side of the axoneme causes it to deform. This deformation bends the axoneme, eventually causing the dyneins to deactivate. Consequently, the dynein motors on the opposing side of the axoneme activate and reverse the bend. Each of these models relies on choosing some parameter that triggers this reversal upon reaching a critical value. In the sliding control model (Murase 1991), there is an elastic resistance of the microtubule doublets to dynein-driven sliding and subsequent bending. The eventual build-up of resistance causes the dynein motors to detach from the neighboring microtubule. Whereas, the curvature control model (Machin 1958; Brokaw 1972; Sartori et al. 2016) relies on the deactivation of dyneins at a threshold value of the curvature of the axoneme, typically understood to take effect with some time delay. Meanwhile, the “geometric clutch” model (Lindemann 1994) relies on the assumption that dyneins are more likely to bind when the interdoublet spacing is below a critical distance, controlled by a transverse force between neighboring doublets.

Instead of treating axoneme bending as the result of an antagonistic relationship between opposing sets of dyneins, models focusing on dynamic instabilities in flexible filaments have garnered interest in recent years. In this model, dynein activity produces a force tangential to the microtubule doublets. In a static filament, this would result in it buckling, however, if this force continually acts along the axis of the filament then the “follower force” can produce oscillatory waveforms in model cilia without the need for individual dynein regulation (Woodhams et al. 2022, and references therein).

### Where are we going?

In this review, we have highlighted the experimental, analytical, and mathematical techniques that can be used to quantitatively characterize microscale motility, allowing measurable descriptions of behavioral dynamics. This enables us to gain insights into how an organism performs in a dynamic environment by overcoming or even exploiting the constraints placed on it by the laws of physics (Wan and Jékely 2021). Quantitative analysis of behavior is also beneficial when comparing different organisms and for using experimental data to test different hypotheses and scenarios, for example, whether motility is beneficial in a turbulent environment at the scale of a cell, or when comparing the efficiency of different swimming mechanisms or the function of complex ciliary arrays (e.g., human airways).

#### Technical challenges

To advance our understanding of microscale motility, two key technical challenges remain. First, although many tracking programs are available, they usually require time-consuming optimization and customization steps to make them applicable to the specific organism of interest. Current tracking methods are most suited to round objects with high contrast. Automated tracking is especially difficult when the object of interest has a time-varying shape. Therefore, we need more general segmentation and tracking algorithms that are applicable to a wide range of morphologies and movement characteristics, while also being simple enough so that they do not require extensive coding experience. In the field of animal behavior, several machine-learning-based algorithms have been developed for tracking animal position and posture (Lauer et al. 2022; Pereira et al. 2022). It would be beneficial to develop similar platforms for tracking the movements of microscopic organisms, including changes in shape and appendage actuation, which is applicable to the wide range of morphologies and does not rely on high-contrast imaging of cells at low density. Machine learning is also emerging as a useful approach to extract meaningful information about spatiotemporal features of cellular motility from imaging data and its potential use in phenotyping motility behavior is an area that could be developed further (Choi et al. 2021). A second technical challenge is data management. When acquiring high-magnification and high-speed videos often required to record microscale motility dynamics, large volumes of data can be accumulated (e.g., an experimental study can generate terabytes of data). Therefore, when planning such experiments, it is crucial to carefully plan the data processing pipeline and to invest in cloud storage or hard drive data management solutions, which can pose financial barriers and require high-performance computing power.

#### Moving beyond model organisms

Most fundamental knowledge about organismal behavior comes from studying model organisms (e.g., *E. coli* for bacteria and *C. reinhardtii* for microalgae), partly because they are amenable to genetic manipulation, which allows testing of specific motility machinery or signaling processes related to behavior. However, model organisms are not representative of the range of behaviors possible for a given motility mechanism and so generalizations can be inaccurate. There is a need to diversify the range of study organisms, which can give new information on how, for example, the morphology or ecology of an organism (i.e., its niche) modifies behavioral patterns. For microswimmers, a repository of swimming kinematics exists as a tool for comparing movement characteristics (Velho Rodrigues et al. 2021). However, such a tool does not exist for surface-based mechanisms (e.g., gliding). With the recent development of genetic tools, such as CRISPR technology, genetic mutants can be created, and used to explore the questions that are traditionally only possible by using model systems. By doing so, we can look at a broad range of related species and assess the similarities arising from evolution but also the differences specific to that organism.

#### Linking different scales

Perhaps one of the biggest challenges is to connect understanding across different length scales, that is, from the mechanics of molecular motors and the locomotor behaviors of individuals to large-scale community processes and biogeochemical cycles. This challenge is not entirely new and has been a prevalent question in behavioral and migration studies of macro-organisms (e.g., insects, birds, whales). A recent framework that attempts to bridge this disconnect is movement ecology, which provides a way to link the physiological and behavioral properties of individuals to movement patterns across spatial and temporal scales (Wisnoski and Lennon 2022). Movement ecology is based on four different factors—the movement mechanism, the internal state of the organism, the navigation and re-orientation capabilities, and the environmental context of the organism. This framework combines insights from cell biology, ecology, and evolution, which has promising potential to synthesize a more thorough understanding of the causes and consequences of locomotion (Wisnoski and Lennon 2022). Additionally, quantitative analysis of experimental data combined with theoretical modeling is a powerful tool for bridging the gap between scales and building a cohesive understanding of behavior. As discussed throughout this review, modeling allows testing/simulating conditions that cannot be explored experimentally and experiments can be used to validate models. An example of this multi-scale approach is in investigating the dynamics of harmful algal blooms by integrating studies on molecular biology, individual and collective organismal behavior (e.g., gyrotaxis and vertical migration), and the physical environment (e.g., turbulence, nutrient availability) through various modeling approaches, in the hopes of improving prediction and forecasting (Berdalet et al. 2014; Franks 2018).

#### Collaborating across disciplines

To successfully elucidate different aspects of microscale motility, specific skill sets and knowledge from varied disciplines need to be combined. Traditionally, the “why” questions of function and evolution might be viewed as the premise of biologists and ecologists, while physicists, mathematicians, and engineers ask the “how” questions of forces and mechanics. The methods of investigating behavior can vary between different fields, hence, generated knowledge is specific to the scale and design of the study. Organismal behavior is multi-faceted in nature, therefore the integration of different techniques and disciplines by working collaboratively can give rise to more thorough insights into the multi-scale aspects of behavior. Although interdisciplinary collaborations already exist, the difficulty lies in the lack of a shared foundation for what is considered common knowledge. Thus, there is a need to simplify communication to enhance the flow of information. An example of such a cross-disciplinary initiative is the “motile active matter roadmap” by Gompper et al. (2020), which brought together researchers from diverse disciplines to assess the current state of the art of the active matter field.

We hope this review can be a starting point and toolkit for researchers looking to describe behavior quantitatively in new and exciting systems. Here, we have highlighted both the limitations and the scope of what can actually be measured from experimental systems to test model predictions, while also identifying the areas where modeling would be particularly useful. The field is ripe for researchers to conduct more quantitative analyses, widen the diversity of study organisms, and collaborate across disciplines to drive real progress in our understanding of the multiscale processes of microscale motility.

## Notes

From the symposium “Large-scale biological phenomena arising from small-scale biophysical processes” presented at the annual meeting of the Society for Integrative and Comparative Biology virtual annual meeting, January 16–March 31, 2023.
